# Ether and Chloroform

**Published:** 1881-02

**Authors:** 


### ETHER AND CHLOROFORM.

In the *Lancet*, November 20, Dr. Saundby has a judicious article on these anæsthetics. He observes that inflammation of the lungs or air passages forbids the use of ether. The vapor of ether irritates healthy lungs, often to an excessive degree, and sometimes causes a slight bronchitis for a day or two, while occasionally it gives rise to fatal œdema of the lungs, even where no previous disease existed in these organs. It is, therefore, plain that all inflammatory conditions of the lungs are likely to be made worse by ether. Chloroform is to be preferred in all such cases. Cardiac disease *per se* does not contra-indicate ether, as the drug aids a weak heart. In aortic incompetence with badly filled arteries the circulation becomes better during the administration of ether. In mitral disease the case is somewhat different. It must be remembered that ether frequently causes spasmodic dyspnœa, which ordinarily need cause no alarm, and calls for nothing but temporary suspension of the administration, but during which there is great venous turgescence, and the right side of the heart is necessarily overloaded with blood. So that wherever the right side of the heart is weak and dilated one should prefer chloroform to ether. The same would hold good of dilatation of the right ventricle apart from mitral disease.

Fractures, herniæ, and other conditions in which complete muscular relaxation is required, are cases in which, *cæteris paribus*, one should use chloroform.

